# Tritium-Labeled Compounds V. Radioassay of Both Carbon–14 and Tritium in Films, With a Proportional Counter^1^

**DOI:** 10.6028/jres.064A.038

**Published:** 1960-08-01

**Authors:** Horace S. Isbell, Harriet L. Frush, Nancy B. Holt

## Abstract

A convenient procedure is described for the radioassay of both carbon-14 and tritium in water-soluble, nonvolatile compounds by means of a windowless, gas-flow, proportional counter. The materials are counted in uniform films of sodium *O*-(carboxymethyl) cellulose that are “infinitely thick” to the radiation of tritium but not to the radiation of carbon-14. Films of uniform thickness are obtained by new techniques which are described in detail.

If only carbon-14 is present, its absolute activity can be calculated conveniently by means of an empirically established curve for the counting-efficiency. If both carbon-14 and tritium are present, the films are counted in the proportional counter and are then recounted in the presence of a screen that stops all radiation from tritium but only a portion of that from carbon-14. From a film with a thickness of 0.8 mg/cm^2^, approximately 43 percent of the radiation of carbon-14 is counted. Of this emerging radiation, approximately 50 percent passes through a screen of ¼-mil double-aluminized “Mylar.” By use of suitable calibration curves for counting-efficiency, carbon-14 and tritium in the same sample can be calculated from the counts with, and without, the screen.

Satisfactory analyses can be made with samples containing less than 0.001 microcurie of carbon-14 and 0.005 microcurie of tritium. The method is suitable for the radioassay of a wide variety of labeled materials.

## 1. Introduction and Discussion

Many chemical reactions can be studied by means of double labeling with radioisotopes, as, for instance, with carbon-14 and tritium. However, maximal use of methods employing carbon-14 and tritium has thus far been hampered by the lack of convenient procedures for determining the two radioisotopes in the same sample. Satisfactory analyses of materials containing both carbon-14 and tritium can be made with scintillation counters, but these are expensive and not generally available.

In a previous report from this laboratory, a convenient procedure was described for the radioactivity assay of tritium in water-soluble, nonvolatile compounds by means of a 2*π*, windowless, gas-flow, proportional counter [1].^2^ The sample to be analyzed is contained in a film of sodium *O*-(carboxymethyl)-cellulose that is “infinitely thick” to the radiation of tritium. It seemed possible that this method might be so modified that it could be used for the radioassay of carbon-14 and, especially, of carbon-14 and tritium in the same sample. This paper reports such a method.^3^

Radiation from carbon-14 has considerably greater energy than that from tritium.^4^ Consequently, it is impracticable to prepare films that are “infinitely thick” to this radiation. Films that are “infinitely thick” only to the radiation from tritium absorb amounts of that from carbon-14 which are dependent upon film thickness. However, it was found that, by means of an experimentally determined curve for counting-efficiency, absolute determinations of carbon-14 in films of sodium *O*-(carboxymethyl) cellulose (CMC) could be made with high efficiency and good accuracy.^5^

In order to determine carbon-14 and tritium in the *same* film, advantage was taken of the fact that the radiation from tritium is completely stopped by a screen that permits passage of a considerable fraction of the radiation from carbon-14. By use of experimentally determined counting-efficiences, carbon-14 and tritium can be conveniently determined in the same film from the counts with, and without, a suitable screen.

The radioactivity of tritium-containing materials decreases significantly in the course of several months, and hence a table of the change in activity with time is included for the convenience of the analyst (see table 4, p. 367). This table is based on a half-life value for tritium of 12.26 yr [7].

## 2. Materials and Apparatus

### 2.1. Radioactive Materials

d-Glucose-*1-C*^14^ was prepared by the method previously described [8] and was standardized by radioassay of a formamide solution of it in a proportional counter [6]. d-Glucose-*1*-*t* [9], was assayed in a proportional counter [10] by comparing its activity in a sulfuric acid-phosphoric anhydride solution with that of the NBS standard tritium oxide sample No. 4926.

### 2.2. CMC Stock Solution

The stock solution used with materials of high specific activity consisted of 1.5 g of sodium *O*-(carboxymethyl)cellulose (CMC),^6^ 0.5 g of anhydrous d-glucose, 0.2 g of phenol, 5 mg of a dye (*p*-rosaniline hydrochloride), and sufficient water to give 100 ml of solution. The solution was conveniently prepared by mixing the materials with slightly less than the desired amount of water and allowing the mixture to stand for 24 hr or longer. When the CMC had dissolved, the addition of water was completed. The added phenol prevented the growth of bacteria and molds, and the dye revealed the degree of uniformity of the film. In the stock solution used with samples of low specific activity, part or all of the d-glucose, which acts as an organic “ballast” material and plasticizer, was omitted.

### 2.3. Equipment for Counting

Most of the radioactivity measurements were made at 2000 v with a commercial, 2*π*, windowless, gas-flow, proportional counter.^7^ The commercial counting gas (consisting of 90% of argon and 10% of methane) was dried, before use, by successive passage through soda-lime, anhydrous calcium sulfate, and phosphoric anhydride.

A few measurements of carbon-14 in films were made by means of a commercial “pancake-type” Geiger-Müller tube,^8^ in order to confirm the applicability of this equipment to the radioassay of carbon-14 in CMC films.

Stainless-steel, cupped, flat-bottomed, counting planchets, 2 in. in diameter, supplied for use with the proportional counter, were modified by enclosing a 5-cm^2^ area in a shallow, circular groove (cut on a lathe) beyond which the CMC solution did not spread.^9^

**Figure 1:**
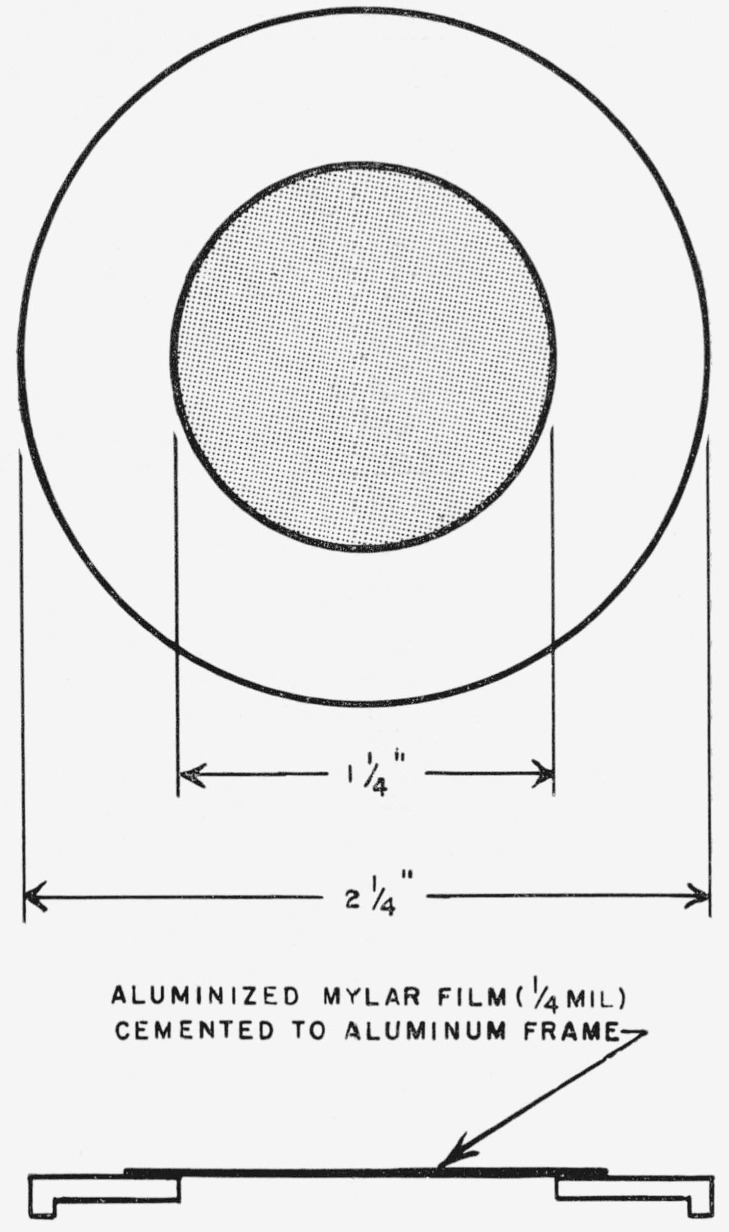
Screen for counting carbon-14 in the presence of tritium.

The screen used for stopping the radiation of tritium, in the radioassay of tritium and carbon-14, is shown in figure 1. Double-aluminized “Mylar” film,^10^ ¼-mil in thickness, and having a weight of of 0.9 mg/cm^2^ was cemented to a circular, aluminum frame that fitted over the top of the planchet. (The thickness of the foil was determined by weighing a sample of known area.) The ability of this foil to stop the radiation from tritium completely was tested by comparing the background count in the absence of radioactive material with that found when the screen shielded a film containing d-glucose-*1*-*t.* The counts were the same, within the statistical error of measurement.

### 2.4. Preparation of Films and Counting of Their Activity

The planchets were polished by rubbing them, immediately before use, with a dry, 3:1 mixture of infusorial earth and Versene [the tetrasodium salt of (ethylenediamine)tetraacetic acid]; excess cleaning powder was removed by means of a stream of air.^11^ This treatment gives a surface on which the liquids used can spread readily.

For every preparation of the CMC stock solution, the exact weight of the total solids per gram of solution was determined by evaporating weighed quantities of the solution (about 1 g), contained in each of several 2-in. planchets, under the conditions used in the analysis. The weight of the residue per gram of the stock solution was used in calculating the weight (*m*) of total solids in the films made from the stock solution and radioactive materials. A known weight of the sample to be analyzed was dissolved in about 2 ml of the CMC stock solution, and the total weight was determined.^12^ A known weight of this radioactive solution (about 200 mg) was carefully spread over the inscribed circle of each of several planchets, cleaned as described above. (These weights are most conveniently determined from the weights of a *200-lambda* pipet, first, filled with the solution, and then, emptied.) Each planchet was covered with an inverted, 6-in. Petri dish 13 and allowed to stand at room temperature overnight. The slow evaporation of the solvent left a uniform film, which was heated under an infrared light for 30 min. It was then conditioned by storing it for at least 1 hr in a desiccator over a saturated solution of potassium acetate (in contact with solid potassium acetate). Any planchet in which the edges of the film deviated from the circumscribed area was discarded.

After being conditioned, the films were counted in the windowless, proportional counter operated at 2,000 v. The counter was flushed with the counting gas for 30 sec at the start, and for 10 sec between the counting periods. After a preliminary counting period of 1 min, which was disregarded, the activity of the sample was counted, for 3 periods of 100 sec or longer, to give a total count of at least 10,000. If the counting rates for the several periods changed progressively, the film was reconditioned by storing it for 1 hr or longer over the saturated potassium acetate; it was then re-counted. The counts were corrected for background (determined by counting a film which had been prepared in the usual manner but which contained no added radioactive material).

### 2.5. Determination of Counting-Efficiencies

**Figure 2:**
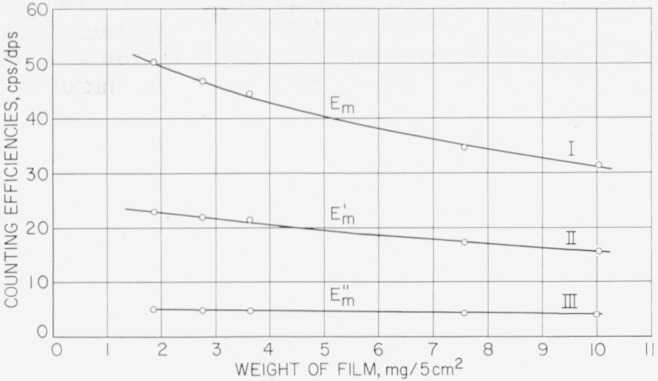
Counting efficiencies for carbon-14 in films. I. Film without screen. II. Film covered with aluminized Mylar screen. III. Film counted with a thin-window, “pancake-type” Geiger-Müller tube.

By means of empirical calibrations of counting-efficiencies, it is possible to calculate the absolute activity of carbon-14 from the count given by a film of known weight. Figure 2 and table 1 show experimentally determined relationships between the counting-efficiency for carbon-14 (i.e., cps/dps) and the weight of the film under the various conditions employed in this laboratory. Each point was determined by counting 5 films, of known weight, each containing a known amount of carbon-14. The films were prepared, as described in section 2.4, from weighed amounts of a radioactive solution containing known amounts of solids per gram. Each planchet was counted by three different methods. The counting-efficiencies of curve I (*E_m_*) were determined with the windowless, gas-flow, proportional counter; those of curve II $\left(E_{m}^{\prime }\right)$ were determined in the same manner, with the film covered by the Mylar screen of figure 1; those of curve III $\left(E_{m}^{\prime \prime }\right)$ were determined with the thin-window, Geiger-Müller tube. For routine analyses, a reference table of values of *E_m_*, $E_{m}^{\prime }$, and $E_{m}/E_{m}^{\prime }$ was prepared from the curves, for use with films of various thicknesses (see table 2). Having once been determined, the values for counting-efficiencies can be used until a change is made in the equipment or procedure. It is advisable to determine counting-efficiencies by the several methods *with the same films.* The ratio $E_{m}/E_{m}^{\prime }$ for a given film-thickness is then independent of the absolute activity in the film.

### 2.6. Determination of Carbon-14

The absolute activity of the carbon-14 in a film is given by the equation,

$$x=\frac{a}{37,000\;E_{m}},$$ (1)

where *x* is the activity in microcuries, *a* is the observed counts per second (cps), and *E_m_* is the appropriate counting-efficiency. If the Geiger-Müller tube is used, the absolute activity of the carbon-14 is likewise calculated from eq (1), but with values for *E_m_*″ There is little difference in the precision of the two methods, but the windowless counter is preferable because it is more sensitive.

### 2.7. Determination of Carbon-14 and Tritium in the Same Sample

In a previous paper, it was shown that the absolute activity of tritium in an “infinitely thick” film may be calculated from the equation,

y=mka, (2)

where *y* is the activity in microcuries, *m* is the weight of the film in milligrams, *a* is the counts per second measured with the windowless proportional counter, and *k* is an empirical constant determined with labeled material of known tritium-content. The factor *k*, for use with the 5-cm^2^ area of the planchet, was determined by the method previously reported. With the equipment used in this study, *k*= 1.94×10^−4^ (with a standard deviation of ±1.6%).^14^

When *both* carbon-14 and tritium are present in the sample, the films are counted in the windowless, proportional counter and are then re-counted after insertion of the Mylar screen (which entirely stops the radiation from tritium but permits passage of a fraction of that from carbon-14). Carbon-14 is then calculated from the equation,

$$x=\frac{a\prime }{37,000E_{m}^{\prime }},$$ (3)

in which *a*′ is the observed count with the screen in place, and $E_{m}^{\prime }$ is the appropriate counting-efficiency. The activity of the tritium present is calculated by the following equation, in which allowance is made for the radiation from carbon-14,

$$y=mk\left[a-a\prime E_{m}/E_{m}^{\prime }\right].$$ (4)

In order to determine the accuracy of the method, a mixture of carbon-14- and tritium-labeled compounds, each of known radioactivity, was analyzed; the results are summarized in table 3. The average amounts of carbon-14 and tritium found differed from the known amounts present by 0.70 percent and 1.65 percent, respectively. The results for thin films and for excessively thick films were less satisfactory than those cited in table 3.

## 3. Comments on the Film-Counting Method

Although the *comparative* counting of carbon-14 in thin layers has long been an accepted and useful technique, general application of the method has not been satisfactory because of uncertainties regarding the thickness of the sample (see ref. [3], p. 29). *Agglomeration* or *crystallization* of materials on the planchet leads to unpredictable variations in the count. The technique described here for the preparation of films has several unique features: (a) The process described for cleaning the planchet produces a surface on which the aqueous solution spreads readily; (b) the use of a thickening agent tends to inhibit crystallization on the planchet; (c) the added coloring matter enables the uniformity of the film to be judged readily; (d) controlled evaporation affords a uniform film; (e) conditioning of the film after drying avoids difficulty (that is otherwise occasionally encountered) with electrostatic charges on the planchet; (f) the presence of “ballast material” in the film makes possible the counting of even highly radioactive materials in films of known thickness. In the range of thickness from 0.4 to 1 mg/cm^2^, good reproducibility and uniformity of the film are readily achieved; whereas, in the extremely thin layers necessary for counting highly radioactive substances *alone*, uncertainties as to film thickness and uniformity are relatively great. With the equipment used, the film-counting method is adequate for determining less than 0.001 *μ*c of carbon-14 or 0.005 *μ*c of tritium.

Hendler [11] has developed, for carbon-14, a linear relationship between an “absorption correction factor” and the weight of the film. Hendler’s relationship is useful for correcting counts, observed at one film-weight, to comparable counts at a desired (reference) film-weight; it also provides an excellent means for detecting errors in the measurement of counting-efficiency by means of the deviation from a linear relationship. The data presented here are in accord with Hendler’s relationship. However, for routine analyses, the *direct* use of counting-efficiencies is adequate and convenient.

## Figures and Tables

**Table 1:** Counting rates of carbon-14 in CMC films

Weight of solution^a^	Activity		Weight of film^b^ (*m*)	Count without screen			Count with screen			
	
				Observed count c (*a*)	Standard deviation^d^	Counting efficiency (*E_m_*)	Observed count^c^ (*a*′)	Standard deviation^d^	Counting efficiency $\left(E_{m}^{\prime }\right)$	$E_{m}/E_{m}^{\prime }$
									
*mg*	*μc*×10^3^	*dps*	*mg*	*cps*	%	*cps/dps*	*cps*	%	*cps/dps*	
100.4	3.851	142.5	1.84	71.7	±2.0	0.5032	32.2	±0.4	0.2259	2.23
150.6	5.777	213.7	2. 75	99.8	0.7	.4670	45.7	.7	.2138	2.18
198.6	7.618	281.9	3.63	125.2	2.0	.4441	58.6	.7	.2079	2.14
154.4^e^	5.738	212.3	7. 57	73.5	1.5	.3462	35.7	.8	.1682	2.06
200.6^e^	7.454	275.8	9.84	85.2	0.9	.3089	42.2	.2	.1530	2.02

a The radioactive solution was prepared by mixing 200.1 mg of an aqueous solution of d-glucose-*1*-*C*^14^ having an activity of 0.964 *μ*c with sufficient CMC stock solution to give a total weight of 25.130 g. The film from 1 g of this solution weighed 18.29 mg and contained 0.003836 *μ*c.

b Each film had an area of 5 cm^2^.

c Average of 5 films at each thickness.

d $\sqrt{\sum d^{2}/\left(n-1\right)}$, where *d*^2^ is the square of the deviation from the mean and *n* is the number of films counted. Results expressed as percent of count.

e d-Glucose was added to the radioactive CMC solution. The film from 1 g of the resulting solution weighed 49.05 mg and contained 0.003716 *μ*c.

**Table 2:** Typical counting-efficiencies for carbon-14 in films, under the conditions described^a^

*m*	*Em*	$E_{m}^{\prime }$	$E_{m}/E_{m}^{\prime }$

*mg*			
3.0	0.461	0.214	2.15
3.1	.457	.213	2.15
3.2	.454	.212	2.14
3.3	.450	.211	2.13
3.4	.447	.210	2.13
3.5	.444	.209	2.12
3.6	.441	.208	2.12
3.7	.438	.207	2.12
3.8	.435	.206	2.11
3.9	.432	.205	2.11
4.0	.429	.204	2.10
4.1	.426	.203	2.10
4.2	.424	.202	2.10
4.3	.421	.201	2.09
4.4	.418	.200	2.09
4.5	.415	.199	2.09
4.6	.413	.198	2.09
4.7	.410	.197	2.08
4.8	.408	.196	2.08
4.9	.405	.195	2.08
5.0	.403	.194	2.08

a Table derived from data such as that of table 1 and fig. 2 in the range of film thickness most commonly used for the analysis of carbon-14 and tritium in mixtures.

**Table 3:** Analysis of a mixture containing known amounts of carbon-14 and tritium^a^

Weight of film (*m*)	C^14^ content	Tritium content	Count without screen (*a*)	Count with screen (*a*′)	$E_{m}^{\prime }$^b^	$E_{m}/E_{m}^{\prime }$ b	*k^c^*	C^14^ found^d^	Diff. from known	Tritium found^e^	Diff. from known
											
*mg*	*μc*×*10*^3^	*μc*×*10*^2^	*cps*	*cps*				*μc*×*10*^3^	%	*μc*×*10*^2^	%
4.50	7.16	5.47	171.0	52.3	0.199	2.09	1.94×10^−4^	7.10^f^	−0.8	5.39^g^	−1.5

a Average of results for 7 films.

b Value taken from table 2.

c See p. 366 and footnote 14.

d Calculated by eq (3), p. 366, from a′ and $E_{m}^{\prime }$.

e Calculated by eq (4), p. 366, from *m*, *a*, *a′*, $E_{m}/E_{m}^{\prime }$, and *k.*

f The standard deviation was ±1.7 percent.

g The standard deviation was ±3.5 percent.

**Table 4:** Decrease of tritium with time^a^

Time	Activity remaining	Time	Activity remaining	Time	Activity remaining
					
*Days*	%	*Days*	%	*Days*	%
0	100.0	250	96.20	500	92.55
10	99.85	260	96.06	510	92.41
20	99.69	270	95.91	520	92.27
30	99.54	280	95.76	530	92.13
40	99.38	290	95.61	540	91.98
50	99.23	300	95.46	550	91.84
60	99.08	310	95.32	560	91.70
70	98.92	320	95.17	570	91.56
80	98.77	330	95.02	580	91.42
90	98.62	340	94.88	590	91.27
100	98.46	350	94.73	600	91.13
110	98.31	360	94.58	610	90.99
120	98.16	370	94.43	620	90.85
130	98.01	380	94.29	630	90.71
140	97.86	390	94.14	640	90.57
150	97.71	400	94.00	650	90.43
160	97.56	410	93.85	660	90.29
170	97.40	420	93.71	670	90.15
180	97.25	430	93.56	680	90.01
190	97.10	440	93.42	690	89.87
200	96.95	450	93.27	700	89.73
210	96.80	460	93.13	710	89.59
220	96.65	470	92.98	720	89.46
230	96.50	480	92.84	730	89.32
240	96.35	490	92.70		

a Based on a half-life of 12.262 yr [7] and the fundamental decay law:
$\ln\;N/N_{0}=-\lambda t.$ (See for example p. 16 of ref. [12].)

## Footnotes

2 Figures in brackets indicate the literature references at the end of this paper.

3 The comparative counting of carbon-14 in films of agar has been reported [2]. The described technique differs considerably from that reported here.

4 The maximum range of the radiation from carbon-14 is about 28 mg/cm^2^ (see ref. [3] p. 7), whereas that from tritium is only about 0.7 mg/cm^2^ [4], A film with a thickness of 28 mg/cm^2^ is difficult to dry to constant weight, and tends to crack and peel. Factors affecting the assay and standardization of *beta*-ray emitters are discussed in ref. [5].

5 This procedure for the counting of carbon-14 supplements the more convenient, but less sensitive, formamide counting-method previously developed in this laboratory [6].

6 Medium-viscosity product, CMC-12MP, of Hercules Powder Co., Wilmington, Del.

7 Model PC-3, Nuclear Measurements Corp., Indianapolis, Ind.

8 No. 1007T of Anton Electronics Laboratory, Inc., Brooklyn, N.Y.

9 For the preparation of films, an area on a planchet, circumscribed by a fine groove, is somewhat less convenient than the 2-in., cupped planchet employed for the counting of tritium only [1]. However, the curved edge of the cupped planchet causes a somewhat uneven distribution of the material. Although unimportant in the counting of tritium, this irregularity in distribution is undesirable in films containing carbon-14, which is counted from *less* than infinite thickness. In the counting of carbon-14 alone, or of carbon-14 and tritium together, the planchets described here should be used.

10 Supplied by Hastings & Co., Inc., Philadelphia, Pa.

11 Rinsing the planchet with water or other solvent impairs the effectiveness of the treatment.

12 Because of the high viscosity of the CMC stock solution, extreme care must be taken to obtain a homogeneous solution with the radioactive material.

13 Controlled evaporation is necessary in order to obtain uniform films. When the planchets were allowed to stand in the open air, the films that formed were thicker in the middle than at the edges; and when the planchets were rather closely covered (with small Petri dishes), the films were thicker at the edges than in the middle. In contrast, the films that formed under the 6-in. Petri dishes were of nearly uniform thickness.

14 Nine films, infinitely thick with respect to the radiation of tritium, and containing 0.01401 *μ*c of tritium per milligram of solids, gave an average count of 72.3 cps. The standard deviation was ±1.09 cps or ±1.6 percent.
